# Detection of reproductive interference between closely related *Salvia* species with small-scale separated distributions by multifaceted pollination and molecular analyses

**DOI:** 10.1007/s10265-024-01577-6

**Published:** 2024-08-30

**Authors:** Sachiko Nishida, Atsuko Takano, Yoshihisa Suyama, Satoshi Kakishima

**Affiliations:** 1 Nagoya University Museum, Furo-Cho, Chikusa-Ku, Nagoya, 464-8601 Japan; 2 University of Hyogo/ Museum of Nature and Human Activities, Yayoigaoka 6, Sanda, Hyogo 669-1546 Japan; 3 Kawatabi Field Science Center, Graduate School of Agricultural Science, Tohoku University, 232-3 Yomogida, Naruko-Onsen, Osaki, Miyagi 989-6711 Japan; 4 Department of Botany, National Museum of Nature and Science, 4-1-1, Amakubo, Tsukuba-Shi, Ibaraki 305-0005 Japan; 5 The Mt. Fuji Institute for Nature and Biology, Showa University, 4562 Kamiyoshida, Fujiyoshida, Yamanashi 403-0005 Japan

**Keywords:** Distributional relationship, Hybridization, Reproductive interference, *Salvia japonica*, *Salvia lutescens*

## Abstract

Reproductive interference, an interspecific interaction in reproductive process that exerts an adverse effect, has gained attention as a contributing factor in promoting exclusive distributions between closely related species. However, detailed studies on the possibility of reproductive interference between native plants are still lacking, presumably because strong reproductive interference can rapidly realize exclusive distributions, leaving the two species apparently independent. *Salvia japonica* and *S. lutescens* are found in separate localities at a small scale, although their distributions overlap at a large scale. We investigated the possibility of reproductive interference between them through field surveys, hand-pollination experiments, evaluation of hybrid fertility, cpDNA and nrDNA genotyping, and genome-wide DNA analysis. The field survey results did not reveal apparent negative interaction in competition for pollinator services. Mixed pollination with conspecific pollen and counterpart pollen reduced seed set in *S. japonica*, and hybrid progeny produced by mixed pollination were less than 20% as fertile compared to the pure species. The DNA genotyping results suggested the possibility of hybridization where their distributions overlap, and the genome-wide DNA analysis results showed clear genetic differentiation between the two species as well as the existence of hybrids. These results suggest that bi-directional reproductive interference between *S. japonica* and *S. lutescens* may have led to their present separated distributions at a small scale.

## Introduction

Reproductive interference, defined as a negative effect of interspecific sexual interactions on the fitness of either species, is one such negative interaction (Gröning and Hochkirch 2008), and it is considered to be a pivotal factor influencing population dynamics, especially between closely related species (Kyogoku 2015). Theoretically, two species that exert strong reproductive interference can achieve exclusive distributions locally (Kuno 1992; Nishida et al. 2015; Ribeiro and Spielman 1986; Yoshimura and Clark 1994) because the species that is less abundant in a locality would suffer from severe interference and produce fewer offspring than the dominant species; in turn, the offspring would suffer from further interference and produce even fewer offspring in the following generations, with the result that the less abundant species would eventually become extinct in the locality.

Hybridization has been proposed as one of the main mechanisms of reproductive interference in plants (e.g. Mitchell et al. 2009), because hybridization can slow the growth rate of a population if hybrid seeds are produced at the expense of conspecific seeds (i.e. interspecific ovule discounting; Levin et al. 1996). Nishida et al. (2020) have suggested that if hybrids occur in a region where only one of the parent plant species is present, we can infer that reproductive interference has led to the exclusion of one of the parent species, unless the hybrids were introduced to the region by a long-distance dispersal mechanism.

**Fig.1:**
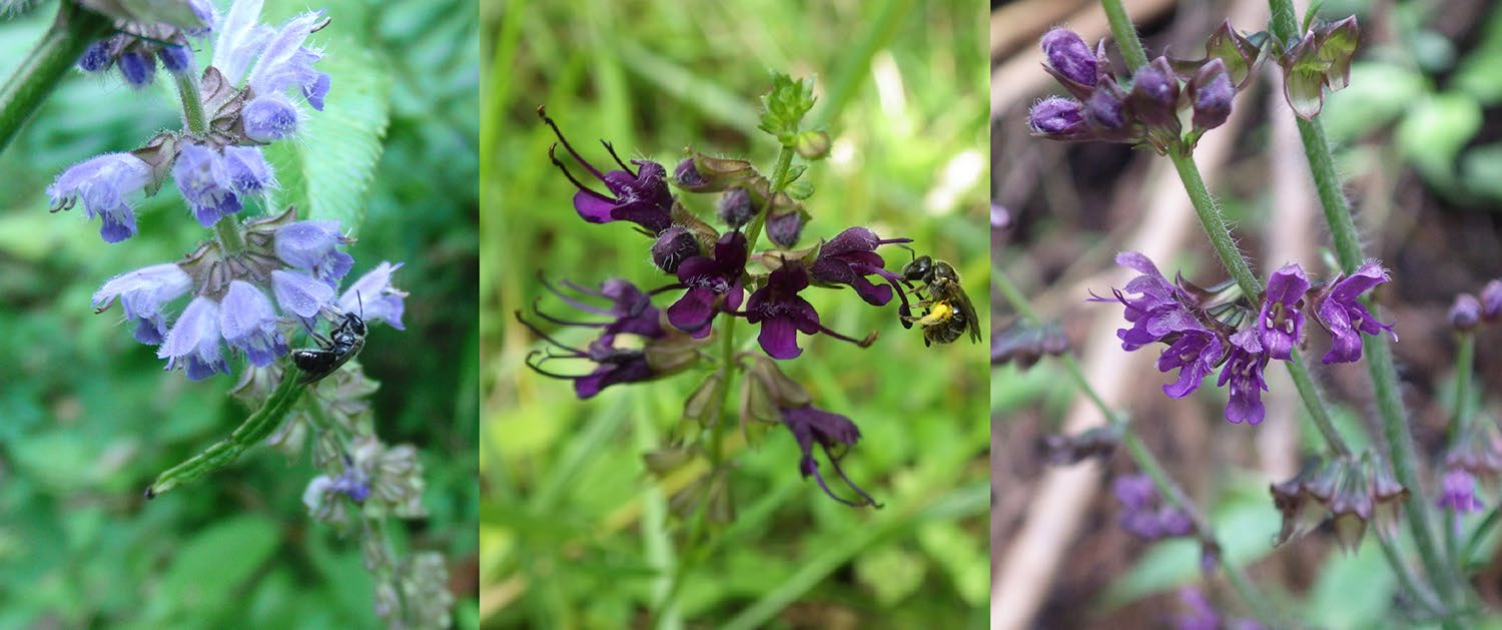
Inflorescence of *S. japonica* (left), *S. lutescens* (middle) visited by a Halictidae bee, and flowers of a putative hybrid between the two species (right)

**Fig. 2:**
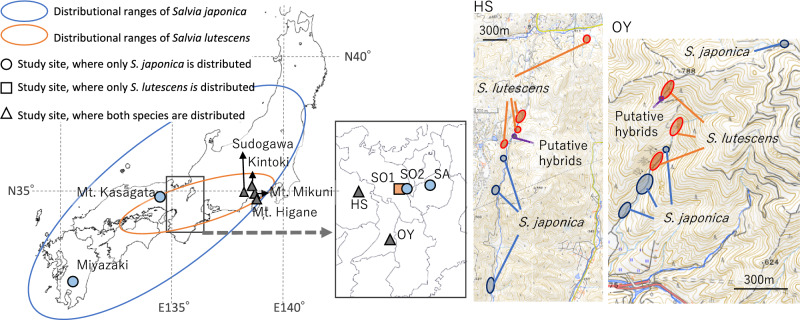
Maps (left) showing the approximate distribution of *S. japonica* (blue oval) and *S. lutescens* (orange oval) in Japan and the study site locations, and maps (right) showing the detailed distribution of each species at HS and OY. SA, SO2, Mt. Kasagata, and Miyazaki (circles) are study sites with only *S. japonica*. SO1 (square) is a study site with only *S. lutescens*. HS, OY, Sudogawa, Kintoki, Mt. Mikuni, and Mt. Higane (triangles) are study sites with both species, according to our preliminary survey on herbarium specimens

With these considerations in mind, we focused here on a pair of wild *Salvia* species that are distributed in overlapping areas, but which occur separately at small scale and investigated the possibility of reproductive interference between them. *Salvia japonica* Thunb. and *S. lutescens* (Koidz.) Koidz. both belong to the subgenus *Glutinaria* (Hu et al. 2018) (Fig. 1). Among the seven species in the subgenus, only these two have overlapping distributions and flowering times in Japan (Takano and Okada 2011). However, at a small scale, their distributions do not overlap (i.e. they are allotopic; Rivas 1964), even when they grow along the same river systems or mountain trails (A. Takano and S. Nishida, pers. obs.; Fig. 2). Takano and Okada (2011) previously suggested that the two species may hybridize, but no detailed study has evaluated this possibility or the possibility that hybridization might be a mechanism of reproductive interference between the two species.

In this study, we carried out fieldwork to investigate whether negative interspecific interactions are presently occurring between these two species, focusing in particular on the possibility of competition for pollinator services. We next conducted a series of hand-pollination experiments and evaluated possible effects of reproductive interference between the species, in particular, the effects of heterospecific pollen on seed set in the two species and on the fertility of offspring produced by hybridization between them. We then studied the two species genetically, using chloroplast DNA (cpDNA) haplotyping, nuclear ribosomal DNA (nrDNA) genotyping, and genome-wide DNA analyses by MIG-seq (multiplexed inter-simple sequence repeat genotyping by sequencing) (Suyama and Matsuki 2015) to detect genetic relationships between them, including hybridization. Through these approaches, we aimed to answer the following questions: Are negative reproductive interactions currently occurring between these species, through hybridization or other mechanisms? Has reproductive interference, including by hybridization, occurred between the two species? Does the present observed small-scale segregation reflect reproductive interference between these two *Salvia* species?

## Materials and methods

### Study species

*S. japonica* and *S. lutescens* are herbs belonging to subgenus *Glutinaria* of genus *Salvia*, Lamiaceae (Hu et al. 2018). In Japan, *S. japonica* is a perennial distributed from Honshu to the Ryukyu Islands (Fig. 2), and it is also found in Korea and China (Murata and Yamazaki 1993). *S. lutescens* is also a perennial, but endemic to the islands of Honshu and Shikoku, Japan (Fig. 2). Both species have similar compound leaves and verticillaster inflorescences, but they differ in flower morphology: *S. japonica* has stamens and styles only slightly longer than the upper corolla lip, whereas the stamens and styles of *S. lutescens* are much longer than the upper corolla lip (Yonekura 2017). The chromosome number of both species is *n* = 16 (Funamoto et al. 2000). Among the varieties of *S. lutescens*, we focused on *S. lutescens* var. *occidentalis* A. Takano in our field survey and hand-pollination experiments. This variety of *S. lutescens* has deep purple (rarely white) flowers, whereas *S. japonica* has pale purple flowers. Although in *S. japonica*, anthesis is reported to last from July to November, and anthesis in *S. lutescens* is reported to extend from June to August (Yonekura 2017), at our study sites, anthesis in both species occurs from July to early September (A. Takano and S. Nishida, pers. obs.).

The two species also share the same insect taxa as pollinators, mainly sweat bees (Halictidae) and hoverflies (*Betasyrphus* and *Bacchini*). These three insect taxa accounted for 89.2% and 86.8% of the total number of pollinator individuals visiting *S. japonica* and *S. lutescens*, respectively, during six observation days (20 July, 3, 8, 13, 27, and 30 August 2019; Y. Watanabe, A. Takano, and S. Nishida, unpublished). Although some *Salvia* species have been reported to be self-compatible (e.g. Haque and Ghoshal 1981; Miyajima 2001), the timing of the male and female stages and the flower structure of the two studied species appear to usually prevent self-pollination: pollen is released from the anthers 1 d before the stigma opens, and the anthers are about 3 mm away from the stigma when the stigma opens (S. Nishida, pers. obs.) Lever-like stamens, though often reported in *Salvia* (Claßen-Bockhoff et al. 2004), are absent in *S. japonica* and *S. lutescens*.

### Study area

Field surveys and cpDNA haplotyping, nrDNA genotyping, and MIG-seq-based single nucleotide polymorphism (SNP) analyses were conducted on populations inhabiting two localities: Sanda, Hyogo prefecture (HS: 35°00′34″N, 135°14′05″E), and Mt. Yamato-Katsuragi, Osaka prefecture (OY: 34°27′11″N, 135°40′14″ E) (Fig. 2). At these two sites, both *Salvia* species were present, but only in small, unmixed patches. The closest distance between individuals of different species was about 100 m at HS and 20 m at OY. All populations at HS and OY were found along rivers and at the edges of forests consisting of planted *Cryptomeria* and wild *Quercus* trees.

To investigate present negative interaction between the two species and for comparison with the populations at HS and OY, we conducted field surveys at Azuchi (SA: 35°09′06″N, 136°08′51″E) and Otsu (SO1: 35°09′34″N, 135°51′52″E), both in Shiga prefecture, as well as cpDNA haplotyping and nrDNA genotyping on each population. The population at SA consisted of only *S. japonica*, and the population at SO1 consisted of only *S. lutescens*. Another isolated population of *S. japonica* at Otsu, Shiga prefecture (SO2: 35°09′15″N, 135°52′06″E), was only about 1.5 km away from the *S. lutescens* population at SO1, but it was confined to a small patch along a road leading to a waste incineration plant. The waste incineration plant and road were built in 1987 or 1988, and we assumed that the *S. japonica* population was introduced after the road was built. On the population at SO2, we performed only cpDNA haplotyping and nrDNA genotyping. We used seeds of *S. japonica* and *S. lutescens* from SA and SO1, respectively, for our hand-pollination experiment and preliminary experiment on survival and conducted these experiments at the Nagoya University Museum Botanical Garden, Nagoya University (35°09ʹ12ʺN.136°57ʹ42ʺE). The garden is within the distributional range of both species, but they do not grow wild at the garden.

We also carried out cpDNA haplotyping and nrDNA genotyping at the following six sites: Miyakonojo, Miyazaki prefecture (31°45′39″N, 130°59′13″E), Mt. Kasagata, Hyogo prefecture (35°03′49″N, 134°50′49″E), Sudogawa, Shizuoka prefecture (35°11′43″N, 138°46′19″E), Kintoki, Kanagawa prefecture (35°16′48″N, 139°00′11″E), Mt. Mikuni, Shizuoka prefecture (35°13′44″N, 138°58′44″E), and Mt. Higane, Shizuoka prefecture (35°08′00″N, 139°03′31″E) (Fig. 2). Among these localities, only *S. japonica* was distributed at Miyakonojo and Mt. Kasagata, whereas both species were distributed at the other four localities, according to data of specimens in the collections of the Museum of Nature and Environmental History, Shizuoka, and the Kanagawa Prefectural Museum of Natural History (A. Takano, pers. obs.). In this study, however, although we found both species at Mt. Mikuni, we found only *S. lutescens* at Mt. Higane, Kintoki, and Sudogawa.

### Negative interspecific interactions in the field

One critical pre-pollination factor that causes negative interspecific interactions is competition for pollinator services (Mitchell et al. 2009). To determine whether the two studied species suffered from pollen limitation as a result of competition for pollinator services between them or with other plants, we compared the seed set following natural pollination with the seed set following conspecific hand-pollination. If either of the species suffers from competition for pollinator services with the counterpart species, the seed set following natural pollination would be lower than that following conspecific hand-pollination especially at the site where both species were present (HS and OY). If they do not suffer from competition for pollinator services with the counterpart species, difference between the seed set following natural pollination and that following conspecific hand-pollination would be marginal. Seed set is the result of many factors, including some abiotic factors such as water, temperature, and nutrients. However, we tried to exclude most factors other than the pollination treatments from our consideration by comparing results under similar conditions within the same study sites. Surveys were conducted at HS in August 2016, at OY in August 2015, at SA in August 2019, and at SO1 in August 2019. In the case of the HS and OY populations, data of seed set following conspecific hand-pollination were collected in the field within each study site. In the case of the SA and SO1 populations, they were collected at the Nagoya University Museum Botanical Garden. At HS, SA, and SO1, we arbitrarily selected approximately 30 individuals of each species at each site and collected one or two fruits from each individual for the data of the natural pollination. At OY, we arbitrarily selected only about 10 individuals and collected about six fruits from each individual because the number of individuals with fruits was limited. The procedure for conspecific hand-pollination is described in the next section. We brought the mature fruits, still in the sepals, to the laboratory and counted the number of normally developed seeds and undeveloped ovules in each fruit. In both species, each flower has four ovules and each fruit has a maximum of four seeds. Normally developed seeds are brown and about 2 mm long, whereas undeveloped ovules are whitish and less than about 0.5 mm long; thus, they are easy to distinguish by eye. We calculated the seed set as the proportion of normally developed seeds relative to the total number of ovules. To statistically evaluate the differences in seed set between natural pollination and conspecific hand-pollination, we used a generalized linear mixed model (GLMM; Wolfinger and O’Connell 1993) with a binomial error structure and a logit link function. The response variable was normal seed development, and the explanatory variable was the pollination treatment. Individuals were incorporated as a random effect. The analysis was conducted with R version 3.5.2 software (R Core Team 2018). We considered the effect to be significant if the *P* value obtained by Wald test was less than 0.05.

### Effect of heterospecific pollen deposition

We conducted hand-pollination experiments to investigate the effect of pollen of the counterpart species on seed set and on hybridization. We used plants from the SA and SO1 populations of *S. japonica* and *S. lutescens*, respectively, because at each of these localities, only one of the two species was present. Therefore, we expected the plants in each of these populations to not have a history of interaction with the counterpart species. The experiment was carried out in September 2019 and July 2021 at the Nagoya University Museum Botanical Garden, where neither species occurs, to avoid the risk of genetic contamination by wild populations. In 2017, we collected seeds of *S. japonica* and *S. lutescen*s at SA and SO1, respectively, and planted each of the seeds in a pot with culture soil at the botanical garden. In 2019, we obtained 12 *S. japonica* plants and 7 *S. lutescens* plants with sufficient flowers for the experiments. We arbitrarily selected about 120 *S. japonica* flowers and 140 *S. lutescens* flowers and assigned them to one of two pollination treatments: conspecific pollination, in which the flowers received only pollen grains of their own species, and mixed pollination, in which the flowers received a mixture of pollen grains of their own and the counterpart species. To avoid unintended pollination by insect pollinators, we brought the plants into a shed next to the garden, where we applied the pollen, and kept them there until the next day, when all the flowers we used had finished flowering. Before each pollination treatment, we first confirmed that there were no pollen grains on the stigma. For pollen donors, we used plant individuals that were not siblings of the recipient plants. We picked up one of the donor stamens with tweezers and applied the donor pollen grains to the recipient stigma by gently brushing the stigma with the donor anther. In the mixed pollination, we applied the conspecific pollen first and the counterpart pollen immediately afterwards. In this way, we avoided overestimation of the effect of the heterospecific pollen on the stigma, which might occur if the counterpart pollen was applied before the conspecific pollen. We did not count the number of pollen grains transferred during each pollination, but we observed a similar number of pollen grains from each species on the stigma after each pollination treatment with a magnifying glass (pollen grains of the two species could be distinguished by their color).

We carried out the same pollination experiments again in July 2021, using both the same plants and the offspring of the plants obtained in 2019 following conspecific pollination. In 2021, we used more individuals of each species (14 *S. japonica* and 19 *S. lutescens*) but fewer flowers (about 30 *S. japonica* and 50 *S. lutescens*) for the experiment compared with that in 2019. We followed the same procedure as in 2019 but avoided using not only siblings but also parent plants as pollen donors.

About 20 days after the hand-pollination in each year, we collected the resulting fruits, brought them to the laboratory, counted the number of normally developed seeds and undeveloped ovules in each fruit, and calculated the seed set. We analyzed the effect of mixed pollination on seed set using a GLMM with a binomial error structure and a logit link function. We analyzed the data in each year both independently and inclusively (i.e., both years together), because insufficient data were obtained in 2021 for independent analysis. In all analyses, the response variable was normal seed development, and the explanatory variable was the pollination treatment. Individual plants were incorporated as a random effect in the independent analyses, whereas they were nested within year before being incorporated as a random effect in the inclusive analyses. The analyses were conducted with R 3.5.2 software (R Core Team 2018). We considered the effect to be significant when the *P* value obtained by Wald test was less than 0.05.

### Hybrid offspring and hybrid offspring fertility

To estimate the frequency of hybridization between the two species, we carried out nrDNA genotyping of the progeny obtained after mixed pollination. We collected all of the seeds obtained from the mixed pollination and sowed each seed in a pot of culture soil. Germination rates were low in both species (17.1% for *S. japonica* and 14.0% for *S. lutescens*) and did not differ significantly between the species (Fisher’s exact test, *P* = 0.46, odds ratio = 0.78). We obtained 20 *S. japonica* seedlings and 44 *S. lutescens* seedlings. We collected one leaf from each seedling, extracted DNA from the leaf, and amplified the internal transcribed spacer (ITS) regions. The procedures for DNA extraction, amplification, and sequencing are described below.

Among those seedlings identified as hybrid, 16 individuals flowered in 2021. We first observed their pollen grains to determine the male fertility. We picked pollen grains from an anther with tweezers, placed them in a drop of distilled water on a concave slide, gently covered them with a cover glass, and observed them under the microscope. The pollen grains of the two species could be divided by their shape into ellipsoid and roundish types. Roundish-type pollen grains were usually less than two-thirds the length of the ellipsoid-type grains and did not stain with potassium iodide, whereas the ellipsoid pollen grains usually stained with potassium iodide (S. Nishida, pers. obs.). Considering the smaller size of the roundish-type grains and the fact that they did not stain with potassium iodide, we inferred that the roundish-type pollen grains had low fertility. We counted pollen grains of both types on each slide from the 16 hybrid individuals, 6 pure *S. japonica* individuals, and 7 pure *S. lutescens* individuals. We calculated the proportion of ellipsoid-type pollen grains to total pollen grains from the hybrid and pure species and analyzed the proportional difference between hybrid and pure individuals using a GLMM with a binomial error structure and a logit link function. In this analysis, the response variable was the pollen grain type and the explanatory variable was the plant type (hybrid or pure species). Individuals were incorporated as a random effect. The analysis was conducted with R version 3.5.2 software (R Core Team 2018). We considered the effect to be significant when the *P* value obtained by Wald test was less than 0.05.

To determine female fertility, we hand-pollinated hybrid individuals. In July 2021, we arbitrarily selected a few whorls of flowers in the verticillaster-type inflorescences of the hybrids and assigned them to one of two pollination treatments: pollination with *S. japonica* pollen and pollination with *S. lutescens* pollen. The pollination procedure was the same as described above for conspecific pollination. About 20 days after the hand-pollination, we counted the number of normally developed seeds and undeveloped ovules in each fruit collected and calculated the seed set. We compared the results with the seed set data for the pure species following conspecific pollination (for details, see the previous section). We analyzed the difference in seed set between the hybrids and pure species using a GLMM with a binomial error structure and a logit link function. The response variable was normal seed development, and the explanatory variable was plant status (hybrid or pure species). Individuals were incorporated as a random effect. The analysis was conducted with R 3.5.2 software (R Core Team 2018). We considered the effect to be significant when the *P* value obtained by Wald test was less than 0.05.

### cpDNA haplotyping, nrDNA genotyping, and assessment of genetic structure using MIG-seq

To detect the occurrence of hybridization, we performed cpDNA haplotyping and nrDNA genotyping of individuals of *S. japonica* and *S. lutescens* and analyzed the genetic structure of the populations in which the two species coexisted using MIG-seq (Suyama and Matsuki 2015).

Leaf samples for cpDNA haplotyping and nrDNA genotyping were collected from all study sites (Fig. 2), but leaf samples for MIG-seq were collected only from study sites HS and OY, where both species were distributed. Most of the individuals sampled at HS and OY were used for all three procedures, cpDNA haplotyping, nrDNA genotyping, and MIG-seq. During sampling, we found two plants at HS and four plants at OY that appeared to be hybrids by their flower morphology, in particular, by their stamen length and petal color, which appeared to be intermediate between those of the two species. We provisionally called these plants putative hybrids.

We extracted total genomic DNA from the dried sampled leaves using a modified version of the 2 × cetyltrimethylammonium bromide (CTAB) extraction protocol of Doyle and Doyle (1987). We amplified the *ycf1–ycf15* region in plastid DNA using 5711f as the forward primer and rps15r as the reverse primer (Drew and Sytsma 2011), and we amplified the nrDNA ITS region using ITS5 as the forward primer and ITS4 as the reverse primer (White et al. 1990). The protocol and conditions for the polymerase chain reaction (PCR), purification, and cycle sequencing analyses followed Takano and Okada (2011) and Takano (2017). Raw sequences were assembled and edited manually using the BioEdit software (ver. 7.2.5; Hall 1999). Multiple DNA sequences were aligned using the multiple alignment method in the CLUSTALW 1.83 software package with default settings (Thompson et al. 1994). Gaps were deleted. For our genotyping, we used the sequences recognized by Takano (2017) as usable for distinguishing between *S. japonica* and *S. lutescens* (Table 1).

**Table 1:** Sequences usable for distinguishing between *Salvia japonica* and *S. lutescens* (extracted from Takano 2017)

Haplotype	Polymorphic allele sites in the ycf1-ycf15 (cpDNA) region						
	89	90	200	228	268		
*S. japonica*	A	G	A	T	C		
*S. lutescens*	C	T	G	G	A		
Polymorphic allele sites in the nrDNA internal transcribed spacer (ITS) region							
Genotype	447	461	464	490	515	580	
* S. japonica*	A	C	C	G	G	C	
* S. lutescens*	G	T	T	A	A	T	

MIG-seq is a PCR-based procedure for constructing highly reduced representation libraries without restriction enzyme digestion steps that involve de novo SNP discovery and genotyping by next-generation sequencing (Suyama and Matsuki 2015; Suyama et al. 2022). For MIG-seq, we mostly used the same extracted DNA that we used for the DNA genotyping. From the DNA extracted from leaves collected from HS and OY, we used 26 and 24 *S. japonica* samples, respectively, 29 and 24 *S. lutescens* samples, respectively, and 2 and 4 putative hybrid samples, respectively. The MIG-seq library was prepared following Suyama and Matsuki (2015). Primer set 1 (Suyama and Matsuki 2015) was used for the first PCR. The number of cycles for the first PCR was set to 25, as Suyama and Matsuki (2015) proposed, but the annealing temperature was decreased from 48 °C to 38 °C to follow the procedure recommended by Suyama et al. (2022). The second PCR products were obtained by using the first PCR products as templates and were purified by using AMPure XP beads (Beckman Coulter, Brea CA, USA). Then, 300–800-bp fragments were isolated using the BluePippin system (Sage Science, Beverly, MA, USA). The final concentrations were measured with a Qubit 3.0 Fluorometer (Invitrogen, Carlsbad, CA, USA) and a 4200 TapeStation (Agilent, Santa Clara, CA, USA). The multiplexed library was sequenced using an Illumina MiSeq Sequencer with MiSeq Reagent Kit v. 3 (150 cycles, Illumina, San Diego, CA, USA) and the dark cycle option, which skipped the first 17 bp of read 1 and the first 3 bp of read 2, following the original protocol (Suyama and Matsuki 2015). As a result, we obtained 80-bp sequences from read 1 and 94-bp sequences from read 2. For quality control of the raw reads, the first 14 bp of read 2 were trimmed using the fastx trimmer program in the FASTX-Toolkit 0.0.14 (https://hannonlab. cshl.edu/fastx_toolkit/). Then both reads 1 and 2 (80 bp each) were trimmed to remove the adapter sequences (GTCAGATCGGAAGAGCACACGTCTGAACTCCAGTCAC and CAGAGATCGGAAGAGCGTCGTGTAGGG AAAGA), the first five bases, the last base, and low-quality regions (quality value [QV] < 15 in a four-base-wide sliding window); short reads (< 74 bases) were removed using Trimmomatic ver. 0.39 software (Bolger et al. 2014). Ipyrad ver. 0.9.90 software was used to assemble the reads and obtain SNP markers (Eaton and Overcast 2020). The depth of coverage was set to six, and the clustering threshold was set to 0.9. Other parameters were set to their default values. The individual-based genetic structure was estimated using the STRUCTURE 2.3.4 program (Pritchard et al. 2000) in the ipyrad analysis toolkit. The sample coverage with the minimum number of SNPs was set to 0.8, and 20 independent Markov Chain Monte Carlo (MCMC) runs with 100,000 iterations were performed, following a burn-in period of 100,000 steps. The number of clusters (K) was set to two under the assumption that there were just two species.

## Results

### Negative interspecific interactions in the field

**Fig. 3:**
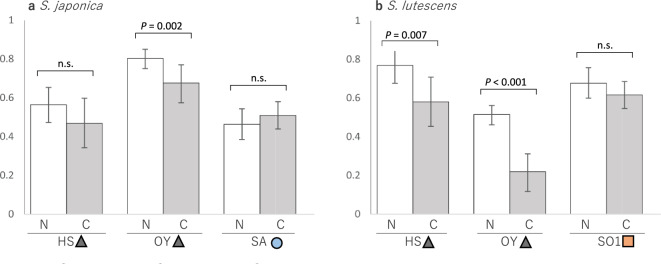
Seed set following natural pollination and conspecific hand-pollination of **a** *S. japonica* and **b** *S. lutescens* at HS, OY, and SA (*S. japonica* only) or SO1 (*S. lutescens* only). N and C indicate natural pollination and conspecific hand-pollination, respectively. Conspecific hand-pollination of the SA and SO1 populations was conducted at the Nagoya University Museum Botanical Garden. A significant effect (*P* < 0.05) of conspecific hand-pollination on seed set was determined by GLMM analyses followed by a Wald test. Error bars show the 95% confidence interval. n.s. = not significant

The seed set of the two *Salvia* species under natural pollination was not significantly lower than that following conspecific hand-pollination at any of the study sites, whether the site harbored both species (HS and OY) or only one of the species (SA, SO1)(Fig. 3).

### Effects of heterospecific pollen deposition

**Fig. 4:**
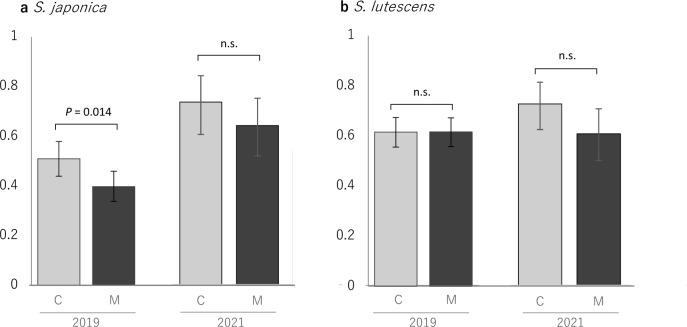
Seed set following conspecific (gray) and mixed (black) hand-pollination of **a** *S. japonica* and **b** *S. lutescens* in 2019 and 2021. A significant effect (*P* < 0.05) of the pollination treatment on seed set was determined by GLMM analyses followed by a Wald test. Error bars indicate the 95% confidence interval. n.s. = not significant

In 2019, seed set of *S. japonica* following mixed pollination was about 22% lower than that following conspecific pollination, whereas seed set of *S. lutescens* was almost the same after conspecific and mixed pollination (Table 2, Fig. 4). In 2021, seed set of both *S. japonica* and *S. lutescens* following mixed pollination was lower than seed set following conspecific pollination, but the adverse effect of mixed pollination was not significant in either species (Table 2, Fig. 4). When we analyzed the results for both years together, the effect was significant in *S. japonica*, but not in *S. lutescens* (Table 2).

**Table 2:** Seed set of *Salvia japonica* and *S. lutescens* following conspecific and mixed hand-pollination and GLMM analysis results for the effect of mixed pollination on seed set

Species	Year	Treatment	*n* (flowers/ individuals)	Seed set	GLMM		
					Coefficient ± s.e	Z	*P*
*S. japonica*	2019	Conspecific	52/12	0.510	−0.506 ± 0.185	−2.733	0.006
		Mixed	66/12	0.398			
	2021	Conspecific	15/13	0.733			
		Mixed	18/14	0.639			
*S. lutescens*	2019	Conspecific	69/7	0.616	−0.159 ± 0.158	−1.005	0.315
		Mixed	73/7	0.616			
	2021	Conspecific	23/19	0.728			
		Mixed	23/18	0.609

### Hybrid offspring and hybrid offspring fertility

The nrDNA alleles of all the pollen recipients we used for the mixed pollination were homozygous and species specific, as shown in Table 1. Of the 20 seedlings obtained from *S. japonica* and the 44 seedlings obtained from *S. lutescens* following mixed pollination, 5 (25%) and 22 (50%), respectively, were identified as hybrids because they were heterozygous in polymorphic loci in the nrDNA region (Type C/D in Table 3).

**Table 3:** Polymorphic sites recognized in *Salvia japonica* and *S. lutescens*

	Number of polymorphic sites in the ycf1-ycf15 (cpDNA) region						
Haplotype	89	90	200	228	268		
Type A	A	G	A	T	C		
Type B	C	T	G	G	A		
Number of polymorphic sites in the nrDNA internal transcribed spacer (ITS) region							
Genotype	447	461	464	490	515	580	
Type C	A	C	C	G	G	C	
Type D	G	T	T	A	A	T	
Type C/D	G/A	T/C	T/C	A/G	A/G	T/C	
Type C/D'	A/G	C	C/T	G/A	G	C	

**Fig. 5:**
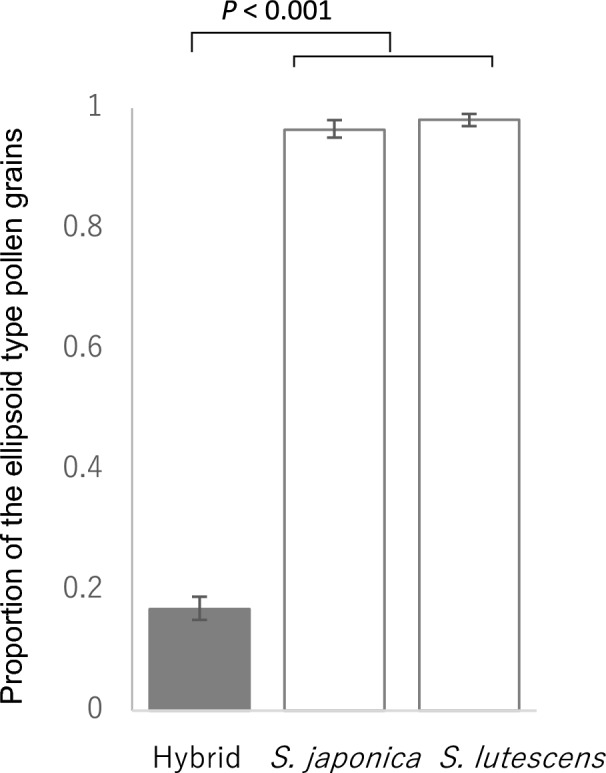
Proportion of ellipsoid-type (i.e., viable) pollen grains in pure individuals of each species and in hybrid progeny. Significant differences (*P* < 0.05) between the hybrids and the pure species were determined by GLMM analyses followed by a Wald test. Error bars indicate the 95% confidence interval

**Fig. 6:**
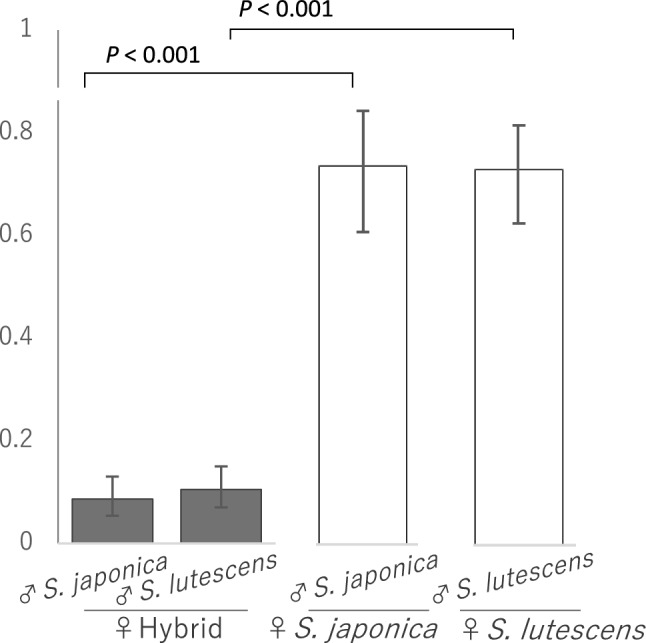
Seed set in hybrid progeny following pollination with *S. japonica* pollen or *S. lutescens* pollen and in pure individuals of each species following pollination with conspecific pollen. Significant differences in seed set (*P* < 0.05) between the hybrids and the pure individuals when pollinated with *S. japonica* pollen or *S. lutescens* pollen were determined by GLMM analyses followed by a Wald test. Error bars indicate the 95% confidence interval

Sixteen of the hybrid offspring flowered in 2021, but the proportion of pollen grains with the normal ellipsoid shape in the hybrids was significantly lower than in the pure species (Fig. 5; GLMM, hybrid coefficient ± SE =  −5.40 ± 0.24, Z = −22.31, *P* < 0.001) and their seed set was lower as well (Fig. 6; GLMM: hybrid coefficient ± SE =  −3.44 ± 0.44, Z = −7.77, *P* < 0.001 for seed set following pollination with *S. japonica*; hybrid coefficient ± SE =−3.16 ± 0.34, Z = −9.20, *P* < 0.001 for seed set following pollination with *S. lutescens*), than seed set of pure *S. japonica* or *S. lutescens* offspring.

### cpDNA haplotyping and ITS genotyping

The cpDNA and nrDNA datasets of 219 individuals (i.e. 88 *S. japonica* and 131 *S. lutescens* individuals) contained 480 and 640 base pairs, respectively, after alignment. The sequences of all haplotypes and genotypes have been deposited in EMBL/GenBank/DDBJ (Accession Nos. LC744806–LC744811). We identified two cpDNA haplotypes, tentatively named types A and B, and four nrDNA genotypes, types C, D, C/D, and C/D’. In cpDNA, five nucleotide substitutions were found between types A and B, and in nrDNA, six nucleotide substitutions were found between types C and D (Table 3). Sequences of types A and C and those of types B and D were confirmed to be identical to the sequences of *S. japonica* and *S. lutescens* recognized in Takano (2017) (compare Tables 1 and 3). In the nrDNA results, types C/D and C/D’ were heterozygous at polymorphic sites (Table 3). The number of individuals of each species from which we were able to obtain haplotypes and genotypes is shown in Table 4. As we explained in the Materials and Methods, we found only *S. lutescens* at Mt. Higane, Kintoki, and Sudogawa during our field surveys, although the specimen data for the museum collections indicated that both species were present at these sites.

**Table 4:** Species presence determined from specimen data, number of individuals of each species found by field survey, and cpDNA haplotypes and ITS genotypes found by molecular analysis of *Salvia japonica* and *S. lutescens*

	*S. japonica*					*S. lutescens*					Putative hybrids			
Locality	Specimen	cpDNA		ITS		Specimen	cpDNA		ITS		Chloroplast		ITS	
		*n*	Haplotypes	*n*	Genotypes		*n*	Haplotypes	*n*	Genotypes	*n*	Genotypes	*n*	Genotypes
HS	Present	26	A	24	C, C/D	Present	29	A, B	29	D	2	B	2	C, C/D
OY	Present	24	A, B	24	C	Present	24	A, B	24	C	4	A, B	4	C
Mt. Mikuni	Present	3	A	3	C	Present	11	A	11	D		–		–
Mt. Higane	Present	–	–	–	–	Present	14	A, B	14	D		–		–
Kintoki	Present	–	–	–	–	Present	15	A, B	15	D		–		–
Sudogawa	Present	–	–	–	–	Present	13	A	12	D, C/D'		–		–
SO1	Absent	–	–	–	–	Present	30	B	30	D		–		–
SO2	Present	10	A	10	C	Absent	–	–	–	–		–		–
SA	Present	11	A	13	C	Absent	–	–	–	–		–		–
Mt. Kasagatake	Present	7	A	7	C	Absent	–	–	–	–		–		–
Miyazaki	Present	10	A	9	C	Absent	–	–	–	–		–		–

**Fig. 7:**
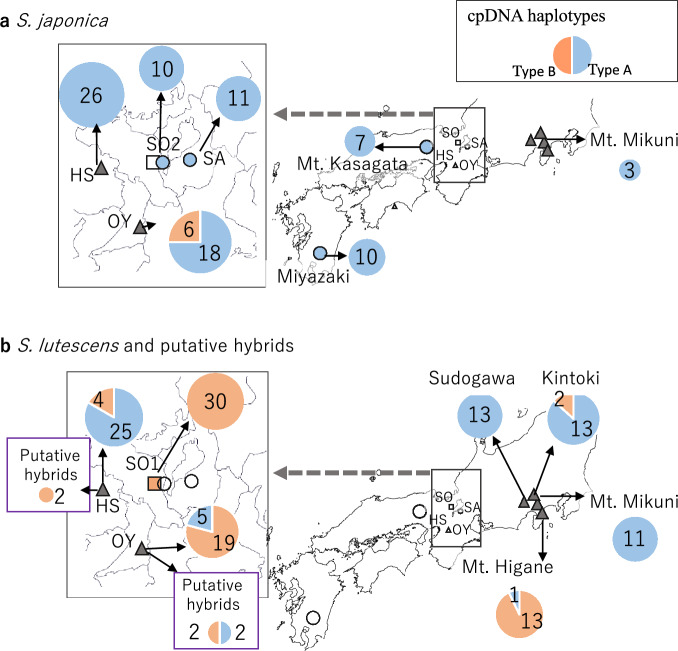
Distributions of chloroplast DNA (cpDNA) haplotypes in **a** *S. japonica* and **b** *S. lutescens* and putative hybrids. The pie chart for each site shows the proportions and numbers of individuals with the type A (blue) and type B (orange) haplotypes. See Table 3 for the nucleotide substitutions between the types

**Fig. 8:**
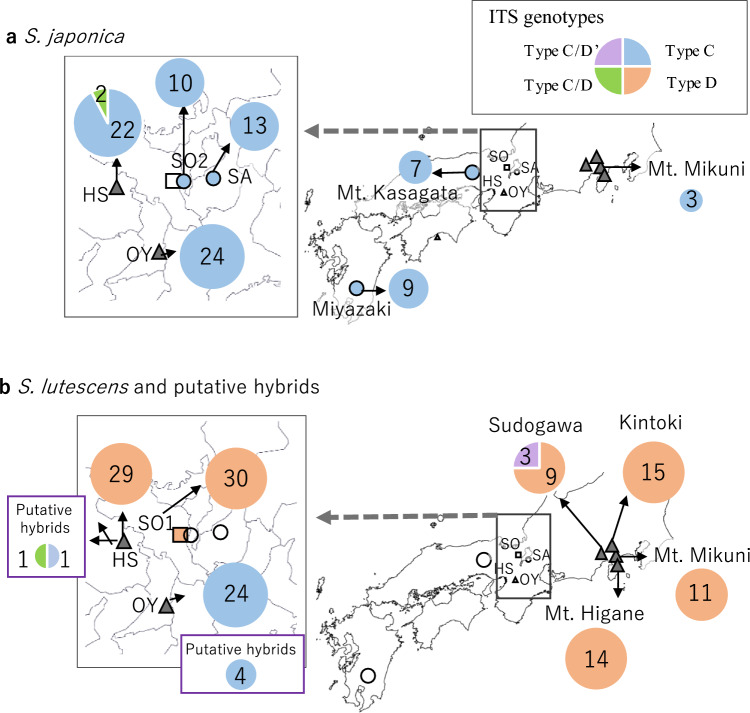
Distributions of internal transcribed spacer (ITS) (nrDNA) genotypes in **a** *S. japonica* and **b** *S. lutescens* and putative hybrids. The pie chart for each site shows the proportions and numbers of individuals with type C (blue), type D (orange), type C/D (green), and type C/D' (purple) genotypes. See Table 3 for the nucleotide substitutions in the types

The haplotypes and genotypes recognized in each locality are summarized in Table 4, and the distributions of haplotypes and genotypes among the sites are shown in Figs. 7 and 8, respectively. Among cpDNA haplotypes, all examined *S. japonica* individuals, except for six individuals from OY, had the type A haplotype (Fig. 7a), whereas at several localities, *S. lutescens* individuals with both type A and type B haplotypes were found (Fig. 7b).

Among nrDNA genotypes, all examined *S. japonica* individuals, except for two individuals from HS, had the type C genotype in the ITS region (Fig. 8a). The two exceptions had the type C/D genotype, in which all polymorphic sites were heterozygous with type C and type D alleles (Table 3). All examined *S. lutescens* individuals, except for the individuals from OY and three individuals from Sudogawa, had the type D genotype (Fig. 8b). All of the OY individuals had the type C genotype, which is the typical *S. japonica* genotype. The three exceptions from Sudogawa had the type C/D’ genotype, in which some of the polymorphic sites were heterozygous with type C and type D alleles and the others were homozygous with type C alleles (Table 3).

Among the putative hybrid individuals, the cpDNA of the two individuals from HS and of two of the four individuals from OY had the type B haplotype, which was the typical *S. lutescens* haplotype (Fig. 7b), and their ITS regions of nrDNA had the type C genotype, which was the typical *S. japonica* genotype, or the C/D genotype (Fig. 8b).

### Assessment of genetic structure by MIG-seq

**Fig. 9:**
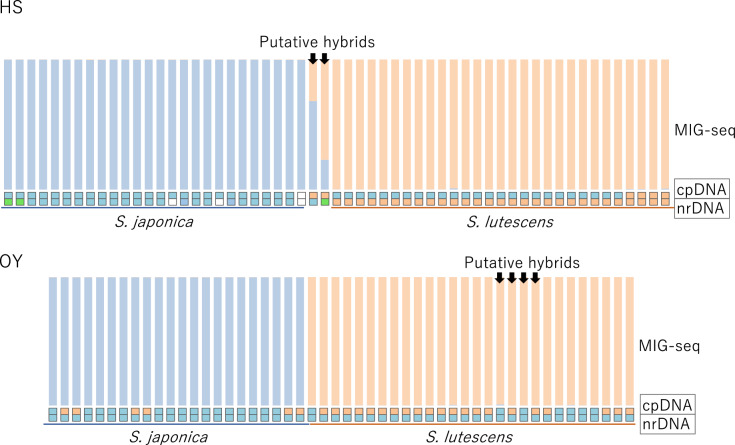
Results of the STRUCTURE analysis of the MIG-seq results for *S. japonica*, *S. lutescens*, and putative hybrids between the two species (arrows) at HS (top) and OY (bottom). The arrangement of the samples roughly corresponds to their distributional relationships (samples near the center are from a locality close to a locality of the counterpart species), except for the two putative hybrid samples at HS, which were from a locality between two *S. lutescens* localities (see Fig. 2). The cpDNA haplotype and the nrDNA genotype of each sample are shown in the small boxes at the bottom (see Figs. 7 and 8 for the types indicated by each color; blank boxes indicate no data)

The total number of reads for all 107 samples following quality control on the raw MIG-seq data was 11,441,713, and the average number of reads per sample was 106,932. After filtering, 147 unlinked SNPs (missing rate, 0.09) were selected and used for Bayesian clustering (STRUCTURE) analyses. Two clusters were recognized with posterior probabilities of > 0.99 that matched morphologically identified *S. japonica* and *S. lutescens*, except for the two putative hybrids at HS (Fig. 9). This result suggests that the two putative hybrids at HS, but not the four putative hybrids at OY, had indeed resulted from recent hybridization between the species. The two hybrids at HS were growing side by side in an area where *S. lutescens* was also growing and about 25 m away from the nearest *S. lutescens* individuals (Fig. 2). One hybrid appeared to be genetically closer to *S. japonica*, whereas the other was genetically closer to *S. lutescens*.

## Discussion

According to our field survey, the two closely related *Salvia* species, *S. japonica* and *S. lutescens*, appear at present to be free of adverse effects from the other species on their reproduction in the wild populations. Our hand-pollination results, however, showed that reproductive interference can occur between the two species: Pollen from the counterpart species could reduce seed set and lead to hybridization producing offspring with significantly low fertility. The cpDNA haplotyping and nrDNA genotyping results suggested that the two species might have a history of hybridization, and our MIG-seq analysis results detected hybrids between the two species.

The number of studies focusing on plants that may be involved in reproductive interference has been increasing (e.g. Briscoe Runquist and Stanton 2013; Brown and Mitchell 2001; Burgess et al. 2008; Eaton et al. 2012; Katsuhara and Ushimaru 2019; Takakura et al. 2009; Takakura and Fujii 2010; Tokuda et al. 2015), and some have used molecular methods to investigate whether any plants were hybrids (e.g. Fei et al. 2020; Fukatsu et al. 2019; Nishida et al. 2020; Takemori et al. 2019). However, to the best of our knowledge, no previous study has investigated the current genetic structure of populations to reveal the current state of species interaction in terms of reproductive interference, as we have done here, by the MIG-seq analysis. Moreover, our study may be one of only a few that have investigated the possibility of reproductive interference between native wild plants through fieldwork, experiments, and genotyping using different regions of DNA and genome-wide SNPs.

Our field survey results showed that seed set after natural pollination (open pollination under natural conditions) was often higher than that after conspecific hand-pollination, whether the two species coexisted (HS, OY) or not (SA, SO1) (Fig. 3). Reasons for the relatively lower seed set after conspecific hand-pollination could be that artificial hand-pollination damaged the flowers and/or that natural pollinators visited the flowers frequently and transported pollen to the flowers more efficiently at some study sites. Considering that in three out of six experiments there was no significant difference between the results after hand-pollination and natural pollination, and that the difference was usually marginal, except for *S. lutescens* at OY, it is unlikely that hand-pollination caused damage to the flowers, but more likely that natural pollinators were more frequent and efficient at some study sites. The results indicate that neither pollen limitation nor conspecific pollen loss are substantial. Pollen limitation and conspecific pollen loss can be caused by competition for pollinator services, for example, if pollinators are attracted by the other plant species and visit the focal plant species less frequently, or if conspecific pollen is wasted on the flowers of the other species, thereby reducing the reproductive success of the focal plant species (Mitchell et al. 2009; Moreira-Hernández and Muchhala 2019; Waser 1978). Considering our results, we inferred that negative interactions between the two species were not at present affecting their seed set.

However, how the separated small-scale distributions of the two species were realized in regions where they co-occur requires explanation (see maps of HS and OY in Fig. 2). At OY, for example, we observed no particular differences between the two species with respect to the altitude or riparian condition of their habitats that could explain their segregation into small patches. In a theoretical study on reproductive interference and niche specialization, Nishida et al. (2015) proposed that under moderate reproductive interference, some habitat segregation or niche specialization between members of a species pair can be expected, whereas under negligible reproductive interference, the two species might coexist locally. Our results from the hand-pollination experiments (Fig. 4) and in the examination of the hybrids (Figs. 5, 6) suggest that some level of reproductive interference, although not detected in the field observations, may have led to their separated distributions at a small scale. According to our results, both species may suffer from bidirectional reproductive interference through reductions of seed set or severe interspecific ovule discounting through the production of infertile hybrids. Ovule discounting (i.e. a reduction in fecundity when hybrid fertilization usurps ovules that would otherwise give rise to non-hybrid (conspecific) offspring; Burgess and Husband 2006; Levin et al. 1996), is an important mechanism of reproductive interference (Mitchell et al. 2009). In the future, other possible reasons, such as resource competition, for the separated distributions should be investigated.

It should be noted that our hand-pollination experiments did not perfectly replicate pollination occurring under natural conditions, because insect pollinators were not involved in our experiments. For example, if pollen from two species is deposited on distinctly separate parts of the pollinator’s body in a manner that prevents contact with the heterospecific stigma, mixed pollination may not occur between some *Salvia* species pairs (Claßen-Bockhoff et al. 2004). If this prezygotic isolation mechanism functions in the two studied species, the adverse effects of reproductive interference would be overestimated by our experiments. However, the pollinators of both *S. japonica* and *S. lutescens* are mainly small bees and hoverflies, and on these pollinators, pollen is not deposited on separate parts of their bodies as it is for larger pollinators such as bumblebees, hummingbirds, and bats (Y. Watanabe, A. Takano, S. Nishida, personal observations). Also, lever-like stamens, which are a key factor in the mechanical isolation of some sympatric *Salvia* species (Claßen-Bockhoff et al. 2004), are absent in the two studied species. Therefore, we suggest that a prezygotic isolation mechanism is unlikely in these species, so the results of our experiments are relevant for evaluating the possibility of reproductive interference between them. In the future, however, experiments with native pollinators should be conducted to examine whether a prezygotic isolation mechanism exists in the studied species.

Our cpDNA haplotyping and nrDNA genotyping results suggest that hybridization may have occurred (Figs. 7, 8). We cannot exclude the possibility that these findings might be caused by polymorphic haplotypes or genotypes originally harbored by each species, especially in the case of *S. lutescens*. However, insofar as each species had only one haplotype/genotype in those localities where it was the only species found, we can reasonably infer that when a haplotype or genotype typical of the counterpart species occurs in the focal species, it indicates some hybridization between the two species.

The STRUCTURE results also showed evidence of hybridization between the species. The two putative hybrids at HS probably resulted from a recent hybridization event (Fig. 9). This result indicates that some hybridization and backcrossing has occurred between the two species, supporting our inference from our cpDNA haplotyping and nrDNA genotyping results that hybridization may have occurred between these species. However, apart from these two hybrids, the STRUCTURE results indicated clear genetic differentiation between the two species (Fig. 9). The markedly low fertility of the hybrids in our experiments suggests that even if hybridization occurs occasionally and the hybrids continue to hybridize with both parent species, no offspring might be found. This result is consistent with our inference that the two species may not coexist for a long time within a patch, despite a history of encounter and interaction between the species in regions where they are co-distributed.

Recently, Kriebel et al. (2019) and Rose et al. (2021) sought to reveal the complicated evolutionary history of *Salvia* species using Anchored Hybrid Enrichment, a DNA sequencing method designed to recover hundreds of unique orthologous loci (i.e., single copy, phylogenetically informative markers) from across the genome and resolve both shallow and deep-scale evolutionary relationships within non-model systems (Hamilton et al. 2016; Lemon et al. 2012). Kriebel et al. (2019), who used a chronogram developed from a super-matrix of genomic data that targeted sequence data from over 500 of the nearly 1000 *Salvia* species, suggested that multiple dispersals of the genus, including *S. japonica* and *S. lutescens*, likely occurred from mainland East Asia to Japan in the Pliocene. Rose et al. (2021) examined data from 179 *Salvia* species retrieved by Kriebel et al. (2019) and quantified the discordance among plastid and nuclear ribosomal loci to investigate whether the discordance could be explained by incomplete lineage sorting or by horizontal gene flow via hybridization and introgression. The results of their multiple analyses suggested that incomplete lineage sorting could not fully explain the observed gene tree discordance, although they could not exclude the possibility of error in the gene tree estimation; thus, horizontal gene flow through hybridization and introgression most likely has influenced both the deep and more recent history of *Salvia*. Considering the findings of Kriebel et al. (2019) and Rose et al. (2021), we think it is reasonable to infer that multiple migrations in the biogeographical history of Japanese *Salvia* species may have led to reproductive interference through hybridization between *S. japonica* and *S. lutescens* at some time in the past. However, further biogeographical analysis with more samples is needed to reconstruct the detailed history of encounters and interactions between these species.

In *Salvia*, a genus famous for its diversity in flower morphology and adaptation to various pollinators (Claßen-Bockhoff et al. 2004), our results showed a possible negative interaction mediated by shared pollinators. Given the species richness of *Salvia* and the wide variation in pollination mechanisms that have been documented for the genus, a number of interesting studies on pollinator syndromes in *Salvia* have recently appeared (e.g. Celep et al. 2020; Wester et al. 2020). Our study, by calling attention to negative interactions, provides an additional perspective on the development of diversity in this genus.

## Data Availability

Data is available in Figshare 10.6084/m9.figshare.24637245.
